# Analysis of the prevalence of and factors associated with overactive bladder in adult Korean women

**DOI:** 10.1371/journal.pone.0185592

**Published:** 2017-09-28

**Authors:** So Young Kim, Woojin Bang, Hyo Geun Choi

**Affiliations:** 1 Department of Otorhinolaryngology-Head & Neck Surgery, CHA Bundang Medical Center, CHA University, Seongnam, Korea; 2 Department of Urology, Hallym University College of Medicine, Seoul, Korea; 3 Department of Otorhinolaryngology-Head & Neck Surgery, Hallym University College of Medicine, Seoul, Korea; University of Pittsburgh School of Medicine, UNITED STATES

## Abstract

**Background:**

Overactive bladder (OAB) is one of the most prevalent lower urinary tract conditions and has been suggested to be related to various factors. We assessed the prevalence of and factors associated with OAB in women based on a large cross-sectional, population-based study of adult Korean women.

**Methods:**

The Korean community health survey (KCHS) of 2012 was reviewed, and 107,950 female participants aged 19 to 107 years were identified for inclusion in this study. The overactive bladder symptom score (OABSS) was used to define and classify OAB as mild, moderate, or severe. Numerous variables, including marital status; physical activity; education and income levels; type of occupation; body mass index (BMI); smoking; alcohol; sleep time; and medical history of hypertension, diabetes mellitus, hyperlipidemia, or cerebral stroke, were evaluated. The correlation of these variables with the prevalence of OAB was analyzed using simple and multiple logistic regression analyses with complex sampling.

**Results:**

The results showed that 5.2% of adult women experienced OAB. Multiple regression analyses showed a significant correlation between the following variables and OAB: older age (adjusted odds ratio [AOR] = 1.44, 95% confidence interval [CI] = 1.39–1.50, P < 0.001 as 10 years older); married status (AOR = 0.83, 95%CI = 0.70–0.96, P = 0.016); lower level of income (AOR = 1.50, 95%CI = 1.34–1.68, P < 0.001); high BMI (AOR = 1.33, 95%CI = 1.23–1.44, P < 0.001); smoking (AOR = 1.24, 95%CI = 1.04–1.47, P < 0.001); long sleep time (AOR = 1.95, 95%CI = 1.69–2.26); and medical history of hypertension (AOR = 1.11, 95%CI = 1.03–1.21, P = 0.011), diabetes mellitus (AOR = 1.38, 95%CI = 1.25–1.53, P < 0.001), hyperlipidemia (AOR = 1.27, 95%CI = 1.16–1.39, P < 0.001), and cerebral stroke (AOR = 2.04, 95%CI = 1.73–2.41, P < 0.001). The level of stress showed a dose-dependent association with OAB (AOR [95%CI] = 3.28 [2.81–3.83] > 2.11 [1.91–2.33] >1.28 [1.16–1.41] for severe > moderate > some stress, respectively, P < 0.001).

**Conclusion:**

The prevalence of OAB was approximately 5.2% among adult Korean women. Older age; high BMI; stress level; sleep duration; levels of income and education; marital status; smoking; and medical history of hypertension, diabetes mellitus, hyperlipidemia, and cerebral stroke were significantly related to OAB in women.

## Introduction

Overactive bladder (OAB) is a condition characterized by lower urinary tract symptoms (LUTS), including an urgency of urination, regardless of accompanied urinary incontinence, frequency, and nocturia [1]. This condition is disruptive, compromises quality of life, and incurs a large economic burden [2,3].

The pathophysiological mechanism of OAB is complicated and still elusive. In both genders, bladder dysfunction and several urinary neurotrophins are the typical urodynamic finding of OAB [4]. Voiding is controlled by complex central and peripheral neural systems. Thus, numerous neurogenic or myogenic etiologic factors may influence overactive bladder [5,6]. Urethral hypersensation may also influence OAB, and several gender-specific differences related to OAB have been suggested. Innate or acquired anatomical and physiological differences in the lower urinary tract may explain gender differences in OAB [7]. The structure of the lower urinary tract is markedly different between women and men. For example, the external urethral sphincter is poorly developed in women. The expression and function of neurotransmitter receptors in the bladder and urethra and micturition patterns also differ between genders [8]. Many women experience childbirth, a series of processes including pregnancy, delivery through the vaginal canal, or caesarean section. In addition, acquired coping strategies for the LUTS are different between women and men [7]. The effects of stress on OAB were suggested to be more common in women [9]. Moreover, fluctuations of ovarian hormones should be considered when discussing OAB in women. Due to these multifactorial origins, therapeutics for OAB are unsatisfactory, with a low treatment outcome and considerable side effects [10].

The overall OAB prevalence has been estimated to be approximately 10–16.6% and is similar in both genders [11–14]. The prevalence of OAB is generally considered comparable between males and females less than 40 years of age and then increases in both genders with increasing age [13,15]. Recently, we reported the prevalence and associated factors of OAB in Korean men [16]. However, gender differences exist in the specific symptom clusters and the clinical impacts of OAB [8]. Approximately half of OAB patients complain of multiple symptoms [12]. In women, storage LUTS are more prevalent than voiding and postmicturition symptoms [7]. For instance, women complain more of urge incontinence [12,14]. Some studies even estimated that OAB symptoms are significantly higher in women, especially OAB with urge incontinence (OAB wet) [17]. Stress urinary incontinence is the most common type in women, while other types of urinary incontinence are more common in men [12]. Furthermore, while resultant OAB symptoms may be similar, the underlying mechanisms of OAB were suggested to be diverse according to gender [13]. Pregnancy, vaginal delivery, body mass index (BMI), diabetes, cognitive impairment, and neurological disorders are predisposing factors for urinary incontinence in women [7]. However, few studies have been conducted to comprehensively review the numerous physical, psychological, and socio-economic variables related to OAB.

The present study was designed to estimate the prevalence of and factors associated with OAB in adult women over a wide range of ages. Using a large population-based study group, the reliability and statistical power were maximized. Furthermore, because multiple pathophysiological factors are involved, we considered numerous variables to investigate the factors associated with OAB.

## Materials and methods

### Study population and data collection

This study was approved by the Institutional Review Board of Korea Centers for Disease Control and Prevention (IRB No. 2012-07CON-01-2C). Written informed consent was obtained from all the participants prior to the survey.

This study was a cross-sectional study that utilized the data from the Korean Community Health Survey (KCHS), which was conducted in 2012. The data were collected by the Centers for Disease Control and Prevention of Korea. The survey gathered information through face-to-face, paper-assisted personal interviews between trained interviewers and respondents. The sample size of the KCHS was 900 subjects in each of 253 community units, including 16 metropolitan cities and provinces. Detailed description including total population and sampling household and participants were reported in KCHS website [16]. The KCHS used a two-stage sampling process. A sample area (tong/ban/ri) was selected in the first stage as a primary sample unit according to the number of households in the area using a probability proportional to the sampling method. In the second stage, the number of households in the selected sample tong/ban/ri was identified to create a household directory. Sample households were selected using systematic sampling methods. This process was used to ensure that the sample units were representative of the entire population [18]. For the sample to be statistically representative of the population, the data collected from the survey were weighted by statisticians based on the sample design (S1 file) [19].

### Survey

A question related to marital status including common-law marriage was included in the survey. To measure physical activity, participants were asked for the number of days spent during the most recent week walking more than 10 minutes. Educational level was divided into 3 groups as follows: uneducated participants and participants who had graduated only from elementary or middle schools were assigned to the “low” education group; graduates of high school comprised the “middle” education group; and junior college graduates, college graduates, and participants in graduate school formed the “high” education group. Occupation was classified into 5 groups according to physical activity level as follows: manager, expert, specialist, clerk; service worker, salesperson; technician, mechanic, production worker, engineer; farmer, fisher, laborer, soldier; unemployed; and student [20]. Participants under 110 cm or 30 kg were excluded from this study. Using criteria for the Asia-Pacific region [21], three BMI (kg/m^2^) groups were generated as follows: low BMI, < 18.5; normal BMI, 18.5–25; and high BMI, ≥ 25. Using the methods recommended by the Organization for Economic Cooperation and Development [22] (i.e., dividing household income by the square root of the number of household members), monthly income was divided by the square root of the number of household members and categorized into 150 lowest (0–840), low-middle (848–1,717), upper-middle (1,732–2,683), and highest (2,687–151 24,000) quartiles. Smoking status was divided into 3 groups: non-smoker, past smoker, and current smoker. The past smokers who had quit smoking for less than 1 year were included in the current smoker group. Alcohol consumption was divided into the following three categories: none; ≤ 1 time a month; 2–4 times a month; and ≥ 5 times a month. Amount of sleep was divided into three groups as follows: ≤ 6 h per day, 7–8 h per day, and ≥ 9 h per day. Sleep hours were surveyed as one hour interval. Participants who slept less than 3 hours per night were excluded from this study. Patients were asked whether they usually felt no stress, some stress, moderate stress, or severe stress. The participants were asked about their histories of other comorbidities, such as hypertension, diabetes mellitus, hyperlipidemia, and cerebral stroke, and participants who reported a history of any of these diseases diagnosed by a medical doctor were recorded as positive.

The overactive bladder symptom score (OABSS), which was developed and validated in the Japanese population, was used in this study [23] (S1 Table). A score ≥ 2 for Question 3 “How often do you leak urine because you cannot defer the sudden desire to urinate?” and an OABSS total score ≥ 3 were defined as having an overactive bladder [24]. Overactive bladder was divided into 3 groups according to the following scores: mild, a total score ≤ 5; moderate, a total score of 6–11; severe, a total score ≥ 12.

### Statistical analysis

The differences in the mean age and number of days walked/week between normal participants (controls) and overactive bladder participants were compared using linear regression analysis with complex sampling. The proportion differences in marriage, education level, occupation, income level, BMI group, smoking, alcohol consumption history, sleep hours, stress level, hypertension, diabetes mellitus, hyperlipidemia, and cerebral stroke history were compared using the chi-square test with Rao-Scott correction.

To identify associations between the related factors and overactive bladder, simple and multiple logistic regression analyses with complex sampling were used. The complex sampling weighting strategy is described in detail in S1 File. In multiple logistic regression, age, number of days walked/week, marriage, education level, occupation, income level, BMI group, smoking, alcohol consumption history, sleep hours, stress level, hypertension, diabetes mellitus, hyperlipidemia, and cerebral stroke were adjusted as cofounders. Two-tailed analyses were conducted, and *P*-values less than 0.05 were considered significant. The adjusted odds ratio (AOR) and 95% confidence interval (CI) for overactive bladder were calculated. All results are presented as weighted values. The results were statistically analyzed using SPSS ver. 21.0 (IBM, Armonk, NY, USA).

## Results

**Fig 1:**
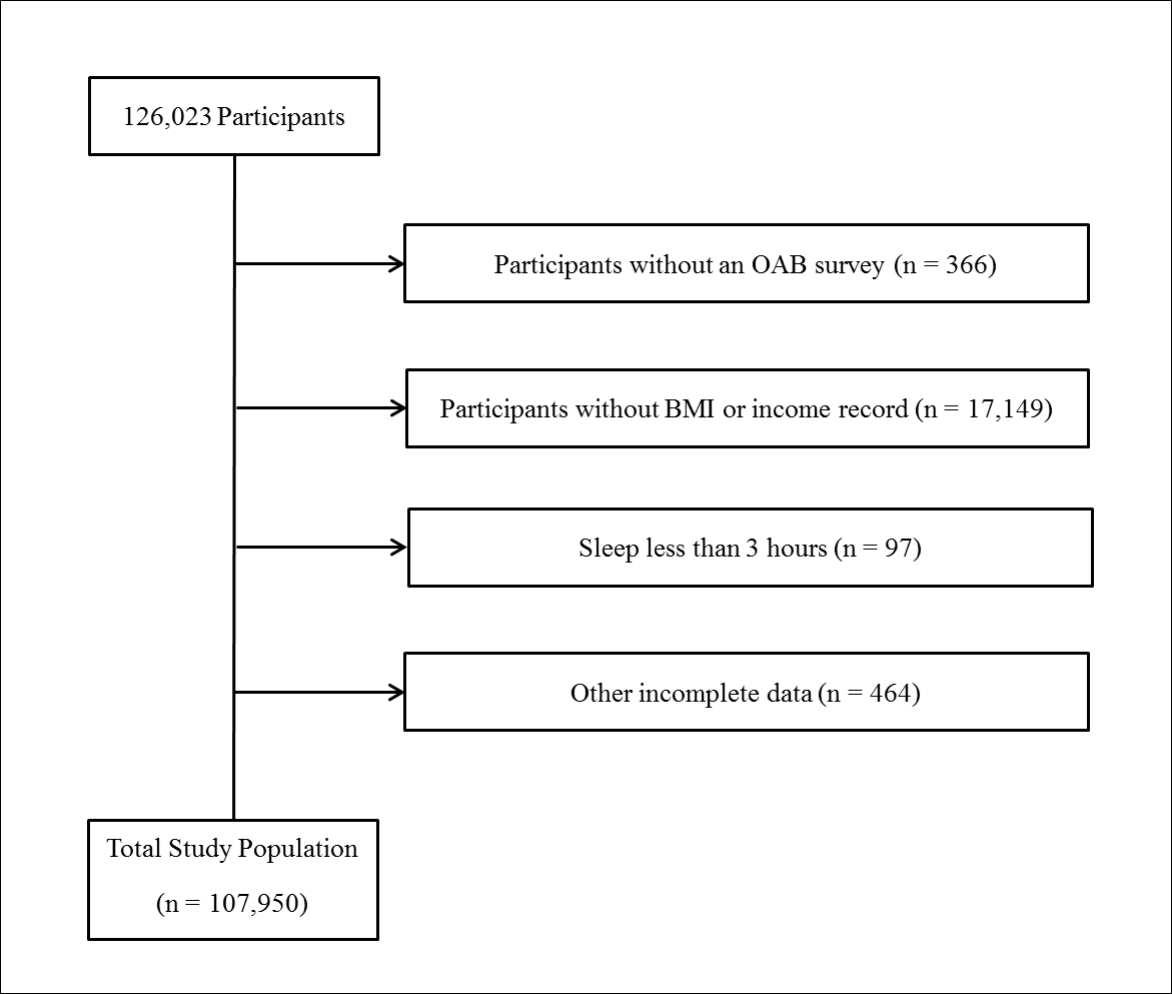
A schematic illustration of participant selection in the present study. Among a total of 126,023 participants, participants without a history of OAB (n = 366), without a BMI or income record (17,149), and with other incomplete data (558) were excluded. The data for the 107,950 participants from whom complete data were obtained were analyzed.

Of a total of 126,023 female participants ranging from 19 to 107 years of age, we excluded the following participants from this study: participants who did not fill out the overactive bladder survey (366 participants); participants who did not indicate height, weight, or income record (17,149 participants); and participants who had incomplete data related to marital status, education level, occupation, smoking, alcohol consumption history, sleep hours, stress level, hypertension, diabetes mellitus, hyperlipidemia, and cerebral stroke (558 participants). Finally, 107,950 participants were included in this study (Fig 1).

**Fig 2:**
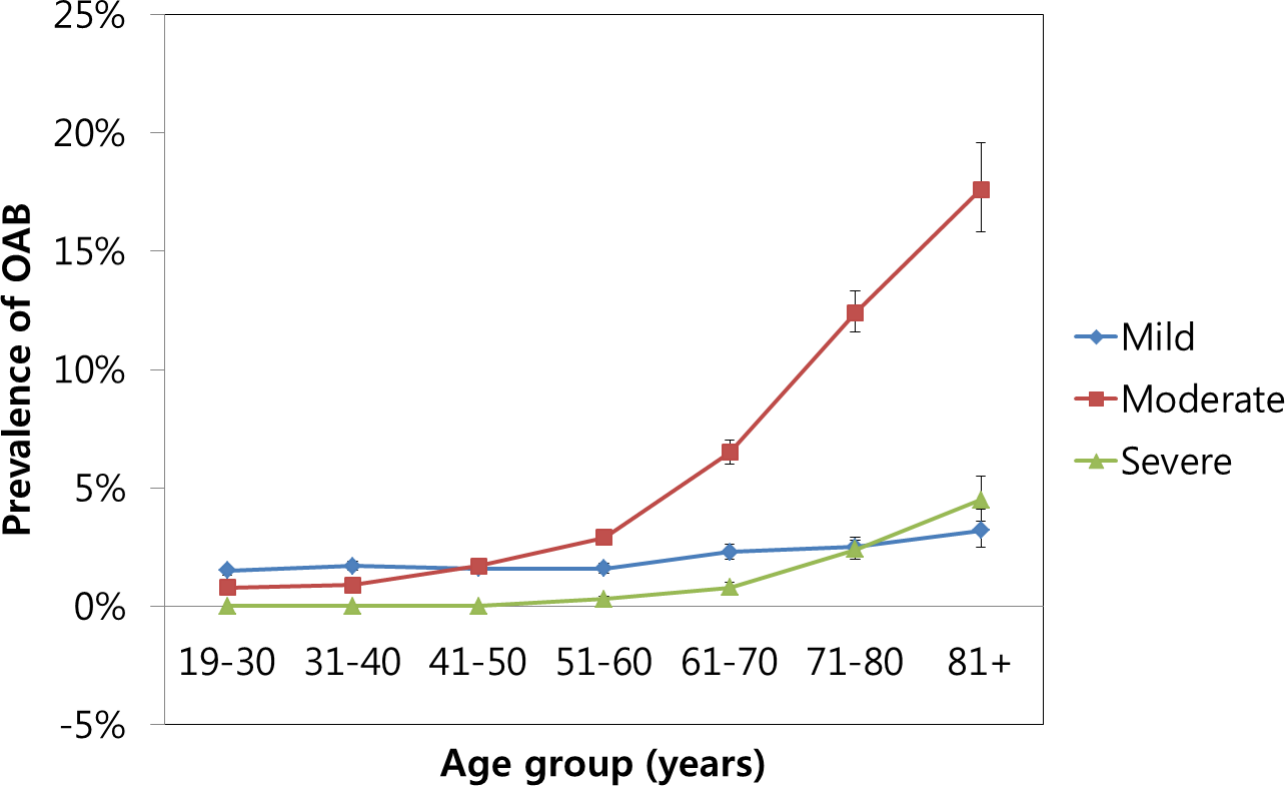
The prevalence of OAB increased with age. According to the severity of OAB, mild, moderate, and severe OAB were relatively comparable from 19–30 years of age through 41–50 years of age and steeply increased after 51 years of age.

Of the 107,950 participants, 6,814 (5.2%) complained of symptoms of OAB. The results showed that OAB increased with age, was lowest in participants aged 19–30 years (2.3%), and was highest in individuals aged 81+ years (25.3%). According to the severity of OAB, the proportions of patients with mild, moderate, and severe OAB were relatively comparable in the 19-30-year-old group through the 41-50-year-old group, and mild OAB was more frequent than moderate and severe OAB. However, moderate OAB steeply increased after 51 years of age (Fig 2, S2 Table). The average age of participants in the OAB group was 58.1 years, which was significantly higher than that of participants in the control group (45.1 years old) (P < 0.001) (Table 1). The OAB group showed significant differences compared with the control group with respect to number of days walked/week; marital status; education level; occupation; level of income; BMI; smoking and alcohol; sleep time; level of stress; and medical history of hypertension, diabetes mellitus, hyperlipidemia, and cerebral stroke (each variable, P < 0.001). All these factors were analyzed for an association with OAB.

**Table 1:** General characteristic of participants.

			Control	Overactive bladder	P-value
Number					
	N		101,136	6,814	
	%, 95% CI‡		94.8 (94.6–95.0)	5.2 (5.0–5.4)	
Age (year, 95% CI)			45.1 (45.0–45.2)	58.1 (57.5–58.7)	<0.001*
Number of days walked/week (d, 95% CI)			4.2 (4.2–4.2)	4.0 (3.9–4.1)	<0.001*
Marriage (%, 95% CI)					<0.001†
	Yes		81.2 (80.9–81.5)	91.2 (91.1–93.0)	
	No		18.8 (18.5–19.1)	7.9 (7.0–8.9)	
Education (%, 95% CI)					<0.001†
	Low		25.8 (25.4–26.1)	57.4 (55.8–59.0)	
	Middle		31.7 (31.4–32.1)	22.0 (20.6–23.4)	
	High		42.5 (42.1–42.9)	20.6 (19.3–22.1)	
Occupation (%, 95% CI)					<0.001†
	Manager, Expert, Specialist, Clerk		22.9 (22.6–23.2)	11.2 (10.2–12.4)	
	Service worker, salesperson		15.5 (15.2–15.8)	11.1 (10.1–12.2)	
	Technician, Mechanics, Production worker, Engineer		2.9 (2.8–3.1)	1.9 (1.5–2.4)	
	Farmer, Fisher, Laborer, Soldier		11.0 (10.8–11.2)	13.1 (12.2–14.0)	
	Student		5.0 (4.8–5.2)	1.3 (0.9–1.8)	
	Unemployed		42.7 (42.3–43.1)	61.4 (59.9–63.0)	
Income (%, 95% CI)					<0.001†
	Lowest		12.1 (11.8–12.4)	31.0 (29.6–32.4)	
	Low-middle		25.7 (25.3–26.1)	28.4 (27.0–29.9)	
	Upper-middle		30.5 (30.1–30.9)	21.3 (20.0–22.8)	
	Highest		31.7 (31.2–32.2)	19.3 (18.0–20.7)	
BMI (kg/m^2^) (%, 95% CI)					<0.001†
	<18.5		9.0 (8.7–9.2)	6.5 (5.8–7.4)	
	≥18.5, <25		73.9 (73.6–74.3)	65.3 (63.8–66.7)	
	≥25		17.1 (16.8–17.4)	28.2 (26.8–29.6)	
Smoking (%, 95% CI)					<0.001†
	None		94.6 (94.4–94.8)	91.3 (90.2–92.2)	
	Past smoker		2.1 (2.0–2.2)	4.0 (3.3–4.7)	
	Current smoker		3.3 (3.2–3.5)	4.8 (4.1–5.5)	
Alcohol (%, 95% CI)					<0.001†
	None		34.6 (34.2–35.0)	52.4 (50.8–54.1)	
	≤ 1 time a month		35.4 (35.0–35.8)	26.2 (24.8–27.7)	
	2–4 times a month		21.5 (21.2–21.8)	13.8 (12.7–15.0)	
	≥ 5 times a month		8.5 (8.2–8.7)	7.5 (6.6–8.5)	
Sleep (hours) (%, 95% CI)					<0.001†
	≤ 6h		43.6 (43.2–44.0)	50.3 (48.7–51.9)	
	7-8h		52.3 (51.9–52.7)	42.0 (40.4–43.6)	
	≥ 9h		4.0 (3.9–4.2)	7.7 (6.9–8.6)	
Stress (%, 95% CI)					<0.001†
	No		15.5 (15.2–15.8)	15.4 (14.3–16.5)	
	Some		57.0 (56.6–57.4)	44.0 (42.4–45.6)	
	Moderate		24.1 (23.7–24.4)	32.8 (31.3–34.3)	
	Severe		3.4 (3.3–3.6)	7.8 (7.0–8.7)	
Hypertension (%, 95% CI)					<0.001†
	Yes		15.1 (14.9–15.4)	36.9 (35.4–38.8)	
	No		84.9 (84.6–85.1)	63.1 (64.4–64.6)	
Diabetes mellitus (%, 95% CI)					<0.001†
	Yes		5.3 (5.1–5.4)	16.4 (15.3–17.6)	
	No		94.7 (94.6–94.9)	83.6 (82.4–84.7)	
Hyperlipidemia (%, 95% CI)					<0.001†
	Yes		10.0 (9.7–10.2)	21.2 (20.0–22.6)	
	No		90.0 (89.8–90.3)	78.8 (77.4–80.0)	
Cerebral Stroke (%, 95% CI)					<0.001†
	Yes		0.8 (0.7–0.9)	4.9 (4.3–5.5)	
	No		99.2 (99.1–99.3)	95.1 (94.5–95.7)	

* Linear regression analysis with complex sampling, Significance at P < 0.05.

† Chi-square test with Rao-Scott correction, Significance at P < 0.05.

‡ CI: confidence interval (weight adjusted).

All the variables showed significant associations with OAB according to simple logistic regression analyses (all, P < 0.001) (Table 2). The prevalence of OAB was significantly increased in older participants (AOR = 1.44, 95% CI = 1.38–1.49, P < 0.001 as 10 years older). More number of days walked/week was significantly related to a decrease in OAB (AOR = 0.99, 95% CI = 0.98–1.00, P = 0.056). The high BMI group was significantly associated with OAB (AOR = 1.33, 95% CI = 1.23–1.44, P < 0.001), and smoking was also significantly related to OAB (AOR = 1.56, 95% CI = 1.28–1.91 for past smoker; AOR = 1.23, 95% CI = 1.03 − 1.46 for current smoker, P < 0.001). A medical history of hypertension (AOR = 1.12, 95%CI = 1.03–1.22, P = 0.009), diabetes mellitus (AOR = 1.38, 95% CI = 1.25–1.53, P < 0.001), hyperlipidemia (AOR = 1.28, 95% CI = 1.17–1.40, P < 0.001), and cerebral stroke (AOR = 2.03, 95% CI = 1.72–2.40, P < 0.001) significantly increased the prevalence of OAB. Compared with the unemployed and student groups, participants in all other types of occupations exhibited a significantly lower prevalence of OAB (P = 0.001). Long sleep time was significantly associated with OAB (AOR = 1.95, 95% CI = 1.69–2.25, P < 0.001).

**Table 2:** Odd ratios of possible risk factors for overactive bladder using simple and multiple logistic regression analysis with complex sampling.

			Simple Regression			Multiple Regression		
			OR	95% CI	P-value	AOR	95% CI	P-value
Age (as 10 years increasing)			1.64	1.61–1.68	<0.001*	1.44	1.38–1.49	<0.001*
Number of days walked/week			0.97	0.96–0.98	<0.001*	0.99	0.98–1.00	0.056
Marriage					<0.001*			0.008*
	Yes		2.69	2.35–3.09		0.80	0.67–0.94	
	No		1			1		
Education					<0.001*			<0.001*
	Low		4.59	4.21–5.01		1.25	1.09–1.43	
	Middle		1.43	1.28–1.59		0.95	0.83–1.08	
	High		1			1		
Occupation					<0.001*			0.001*
	Manager, Expert, Specialist, Clerk		0.34	0.30–0.38		0.92	0.80–1.07	
	Service worker, salesperson		0.50	0.45–0.56		0.84	0.74–0.94	
	Technician, Mechanics, Production worker, Engineer		0.44	0.34–0.57		0.73	0.56–0.95	
	Farmer, Fisher, Laborer, Soldier		0.83	0.76–0.90		0.84	0.77–0.92	
	Student		0.18	0.13–0.25		0.77	0.54–1.11	
	Unemployed		1			1		
Income					<0.001*			<0.001*
	Lowest		4.20	3.82–4.62		1.49	1.33–1.67	
	Low-middle		1.81	1.64–2.00		1.13	1.01–1.26	
	Upper-middle		1.15	1.03–1.28		1.03	0.92–1.16	
	Highest		1			1		
BMI (kg/m^2^)					<0.001*			<0.001*
	<18.5		0.83	0.72–0.94		0.98	0.85–1.12	
	≥18.5, <25		1			1		
	≥25		1.87	1.74–2.01		1.33	1.23–1.44	
Smoking					<0.001*			<0.001*
	None		1			1		
	Past smoker		1.97	1.64–2.37		1.56	1.28–1.91	
	Current smoker		1.48	1.26–1.74		1.23	1.03–1.46	
Alcohol					<0.001*			0.003*
	None		1			1		
	≤ 1 time a month		0.49	0.45–0.53		0.89	0.82–0.97	
	2–4 times a month		0.42	0.38–0.47		0.98	0.88–1.10	
	≥ 5 times a month		0.58	0.51–0.67		1.14	0.98–1.33	
Sleep (hours)					<0.001*			<0.001*
	≤ 6h		1.44	1.34–1.54		1.04	0.97–1.12	
	7-8h		1			1		
	≥ 9h		2.38	2.09–2.71		1.95	1.69–2.25	
Stress					<0.001*			<0.001*
	No		1			1		
	Some		0.78	0.71–0.85		1.28	1.16–1.41	
	Moderate		1.37	1.25–1.51		2.12	1.92–2.35	
	Severe		2.29	1.99–2.64		3.31	2.83–3.86	
Hypertension					<0.001*			0.009*
	Yes		3.28	3.07–3.51		1.12	1.03–1.22	
	No		1			1		
Diabetes mellitus					<0.001*			<0.001*
	Yes		3.53	3.22–3.86		1.38	1.25–1.53	
	No		1			1		
Hyperlipidemia					<0.001*			<0.001*
	Yes		2.44	2.25–2.64		1.28	1.17–1.40	
	No		1			1		
Cerebral Stroke					<0.001*			<0.001*
	Yes		6.43	5.53–7.48		2.03	1.72–2.40	
	No		1			1		

* Significance at P < 0.05.

Adjusted variables in multiple logistic regression: Age, number of days walked/week, marriage, education, occupation, income, BMI, smoking, alcohol, sleep, stress, hypertension, diabetes mellitus, hyperlipidemia, and cerebral stroke.

Level of stress was significantly associated with OAB in a dose-dependent manner (AOR [95% CI] = 3.31 [2.83–3.86] > 2.12 [1.92–2.35] >1.28 [1.16–1.41] for severe > moderate > some stress, respectively, P < 0.001). Subjects with a low education level showed an OAB prevalence that was 1.25-fold greater than that of participants with a high education level (95%CI = 1.09–1.43, P < 0.001). No significant difference was observed in the prevalence of OAB between individuals with middle and high education levels. The prevalence of OAB was significantly higher in the lowest income group (AOR = 1.49, 95% CI = 1.33–1.67) and low-middle income group (AOR = 1.13, 95% CI = 1.01–1.26) than the highest income group (P < 0.001).

Some variables showed different associations with OAB when analyzed using simple vs. multiple logistic regression analyses. Being underweight was significantly associated with OAB in the simple regression analysis (OR = 0.83, 95% CI = 0.72–0.94, P < 0.001). However, the statistical significance was not maintained in the multiple regression analysis. Alcohol consumption showed a negative association with OAB in the simple logistic regression analysis (P < 0.001), but no significant association existed in the multiple regression analysis. Although short sleep time showed a significant association with OAB in the simple logistic regression analysis (OR = 1.44, 95% CI = 1.34–1.54, P < 0.001), this variable was not significantly associated with OAB in the multiple regression analysis.

Marital status showed opposite results between the simple and multiple regression analyses. Married subjects had a significantly higher OAB prevalence according to the simple logistic regression analysis (OR = 2.69, 95% CI = 2.35–3.09, P < 0.001). However, they showed a significantly lower OAB prevalence after adjustment for other variables (AOR = 0.80, 95% CI = 0.67–0.94, P = 0.008).

## Discussion

In the present study, 5.2% of adult women had OAB. This percentage is somewhat lower than that obtained in previous studies, which ranged from 10 to 16% in the ≥ 40 years population based on the self-reported questionnaire on frequency, urgency, and urge incontinence [13,14]. Because our study participants ranged from 19 to 107 years of age and the proportional random selection was conducted with complex sampling, a much younger population may have been included in the present study than in previous studies. In addition, we used the standardized OABSS questionnaire instead of a simple question to assess the presence of OAB symptoms. Several physical factors, such as advanced age; high BMI; and a medical history of hypertension, diabetes mellitus, hyperlipidemia, and cerebral stroke, were significantly associated with OAB according to the multiple logistic regression analyses. Additionally, socio-economic factors, including lower educational and income levels, occupational status of unemployed or student, and being unmarried, lifestyle characteristics of fewer number of days walked/week and smoking, and psychological factors of long sleep time and higher stress level were significantly associated with OAB in the multiple logistic regression analyses. Many of these associate factors of OAB including self-reported stress, obesity, lack of exercise, and lower socioeconomic status are all markers of poor tolerance of homeostatic challenges. These findings were in accordance with previous studies. However, by considering numerous variables, we identified some different results with other studies after adjusted confounders. For instance, marital status increased OAB in the simple logistic regression analysis, while it showed a significantly negative association with OAB after adjustment for other variables.

Aging processes accompany senescence and deregulation of bladder function by diminished muscular and neurological activities [5,6], especially in women who have experienced traumatic obstetric events, including childbirth, and changes in sex hormone levels, which are known to perturb micturition function through related receptors expressed in the lower urinary tract [25]. In this study, these changes during the menopausal period were represented by a marked increase in OAB after 51 years of age.

Obesity was suggested to be a factor that was significantly associated with OAB in several studies, including the present study [13,26]. This association between obesity and OAB can be explained by mechanical and neuroendocrine factors [27]. Adipose tissue can increase autonomic nervous activity, especially that of noradrenergic sympathetic nerves, via leptin production [28]. The resultant increased sympathetic activity causes urinary frequency.

These types of disturbances of the autonomic nervous system may also be related to OAB in the several medical comorbidities associated with obesity. Our results showed significant associations between OAB and a medical history of hypertension, diabetes mellitus, hyperlipidemia, and cerebral stroke. Similarly, previous studies also suggested that obesity-related comorbidities, including metabolic syndrome, diabetes mellitus, and obesity, were linked to OAB [29–31]. In addition to the neural mechanisms, increased ischemia of the bladder in cardiovascular diseases can lead to the overactivity and structural changes of it [32].

In our study, stress showed significant dose-dependent relationships with OAB. Similarly, previous studies demonstrated that several psychological disorders, including anxiety, depression, and stress, are often accompanied by OAB [33,34]. These psychological associations can be explained by the fact that bladder function is regulated by various neural systems, including central nervous system pathways. [35]. A hormonal influence may be involved in OAB. [36,37]. Moreover, women are more likely to be distressed by frequency of urination than men [12]. They are more vulnerable to affective disorders, which may induce hormonal dysregulation. The significant association between OAB and long sleep time is presumed to be mediated by stress or other psychological factors that are related to inadequate sleep duration.

Fewer numbers of days walked/week was significantly associated with OAB. A sedentary posture may have detrimental effects on micturition, as suggested for sitting on the toilet and micturating without pelvic relaxation in women [7]. Immobility may result in a greater physical effort for micturition, which may increase the risk of incontinence [7]. In addition to these physical impacts of sedentary behavior, some psychological factors, such as anxiety and stress, may influence the prevalence of OAB in these groups [33,34].

In contrast to our results, a recent study reported that compared with unemployed women, working females were at a significantly higher risk of OAB [38]. They suggested that detrimental conditions in the work place, including poor hygiene, a dangerous job associated with accidents, uncomfortable posture, carrying heavy weights, and stress, may be attributed to an increase in OAB in working women [38]. However, we included students in the unemployed group and subdivided employed groups according to their job-related physical activities, while the previous study adopted a binary classification of employed versus unemployed. The prevalence of the OAB-related factors of sitting posture and stress was predicted to be high in the unemployed or student group due to exam preparation for university entrance or job searching. Furthermore, reverse causal relationships are possible between the unemployed and OAB. The symptoms of OAB were suggested to be associated with a decrease in work productivity and a higher unemployment proportion [39–41]. Thus, physically compromising conditions, including OAB, could affect occupational status.

Similar to previous studies, a poor socio-economic status with lower education and income levels was related to OAB in this study [40,42]. Socioeconomic disparities in healthy behavior and health status may cause more OAB in lower socioeconomic conditions [43]. Inversely, it is also possible that being unemployed or having a physically compromised status because of OAB results in a low socio-economic status. Smoking was significantly related to OAB in the present study. Several previous studies also demonstrated the increased prevalence of OAB among smokers [44–46]. Smoking may have detrimental effects on pelvic floor function, thereby inducing urinary incontinence [47]. Moreover, smoking was related to bladder overactivity through nicotinic acetylcholine receptor dysfunction in the peripheral and central nervous systems [48]. Moreover, an increase in the occurrence of OAB in women compared with men was associated with smoking [49]. The antagonistic effect of nicotine on estrogen in women may precipitate nicotine-associated bladder overactivity and incontinence [44,49].

Interestingly, unmarried status was significantly related to OAB in the present study. As mentioned previously, this finding was contradictory to the prediction from previous studies. Obstetrical events, such as pregnancy and vaginal delivery, are natural risk factors for urinary incontinence in women [7,50]. However, it was suggested that this increased vaginal delivery-related risk of OAB decreased over time following delivery [26]. Moreover, sex hormonal influence may be related to a decrease in OAB in married subjects. Micturition is known to be regulated by sex hormones based on evidence of the expression of estrogen-inducible progesterone in the female urethra [25]. Sex hormonal changes, such as those that occur during menopause, may be more common in unmarried individuals who have less exposure to sexual activity. Menopause was reported to occur earlier in unmarried, divorced, or widowed women [51].

The present study has several advantages over previous studies. We had a large population-based study group encompassing a broad age range. Additionally, the survey was conducted with a validated symptom score instrument. Not confined to a binary measure of the presence or absence of OAB symptoms, we investigated the degree of OAB symptoms. Information on a large number of potentially confounding lifestyle and medical factors was collected and considered. Importantly, we demonstrated the association of several female gender-specific factors with OAB. However, the present study was based on subjective self-reported OAB symptom scores, which lack objective measures for LUTS. The cross-sectional study design limited the interpretation of the results with respect to identifying causal relationships. A future study with a prospective study design will address the current limitations.

The prevalence of OAB among adult Korean women was approximately 6.3%. Numerous physical, psychological, and socio-economic factors were related to OAB. In accordance with previous studies, older age, high BMI, medical comorbidities, stress, smoking, and levels of income and education were positively related to OAB. In addition, inadequate and long sleep duration, types of occupations, especially unemployed or student status, and unmarried status were related to OAB.

## Supporting information

S1 File The analytic methods of weighting. (DOCX)

S1 Table Overactive bladder symptom score. (DOCX)

S2 Table Prevalence of OAB according to severity. (DOCX)
