# Supplementary material for: Analysis of the prevalence of and factors associated with overactive bladder in adult Korean women
Source: PLoS One. 2017 Sep 28;12(9):e0185592. doi: 10.1371/journal.pone.0185592 (PMC5619804; doi:10.1371/journal.pone.0185592)
Supplement: S1 Table — (DOCX) [file pone.0185592.s002.docx]

**S1 Table** Overactive bladder symptom score

| Question | Frequency | Score |
| --- | --- | --- |
| How many times do you typically urinate from waking in the morning until sleeping at night? | ≤ 7 | 0 |
|  | 8-14 | 1 |
|  | ≥ 15 | 2 |
| How many times do you typically wake up to urinate from sleeping at night until waking in the morning? | 0 | 0 |
|  | 1 | 1 |
|  | 2 | 2 |
|  | ≥3 | 3 |
| How often do you have a sudden desire to urinate, which is difficult to defer? | Not at all | 0 |
|  | Less than once a week | 1 |
|  | Once a week or more | 2 |
|  | About once a day | 3 |
|  | 2–4 times a day | 4 |
|  | 5 times a day or more | 5 |
| How often do you leak urine because you cannot defer the sudden desire to urinate? | Not at all | 0 |
|  | Less than once a week | 1 |
|  | Once a week or more | 2 |
|  | About once a day | 3 |
|  | 2–4 times a day | 4 |
|  | 5 times a day or more | 5 |
