# Supplementary material for: Analysis of the prevalence of and factors associated with overactive bladder in adult Korean women
Source: PLoS One. 2017 Sep 28;12(9):e0185592. doi: 10.1371/journal.pone.0185592 (PMC5619804; doi:10.1371/journal.pone.0185592)
Supplement: S2 Table — (DOCX) [file pone.0185592.s003.docx]

S2 Table Prevalence of OAB according to severity

| OAB | Age (years old) | | | | | | |
| --- | --- | --- | --- | --- | --- | --- | --- |
|  | 19-30 | 31-40 | 41-50 | 51-60 | 61-70 | 71-80 | 81+ |
| No (n, %, 95% CI*) | 13,875  (97.7, 97.4-98.0) | 18,397  (97.4, 97.1-97.6) | 21,283  (96.7, 96.3-97.0) | 20,986  (95.2, 94.8-95.6) | 14,878  (90.4, 98.8-91.0) | 9,452  (82.7, 81.7-83.7) | 2,265  (74.7, 72.5-76.8) |
| Mild (n, %, 95% CI*) | 194 (1.5, 1.3-1.7) | 284 (1.7, 1.5-1.9) | 334 (1.6, 1.4-1.8) | 367 (1.6, 1.4-1.8) | 352 (2.3, 2.0-2.6) | 281 (2.5, 2.2-2.9) | 77 (3.2, 2.5-4.1) |
| Moderate  (n, %, 95% CI*) | 108  (0.8, 0.6-1.0) | 175  (0.9, 0.8-1.1) | 329  (1.7, 1.5-2.0) | 691  (2.9, 2.7-3.2) | 1,101  (6.5, 6.0-7.0) | 1,399  (12.4, 11.6-13.3) | 515  (17.6, 15.8-19.6) |
| Severe  (n, %, 95% CI*) | 0 (0.0) | 13 (0.1, 0.0-0.1) | 17 (0.0, 0.0-0.1) | 51 (0.3, 0.2-0.4) | 148 (0.8, 0.7-1.0) | 251 (2.4, 2.0-2.8) | 127 (4.5, 3.6-5.5) |

*Estimated prevalence and 95% Confidence interval
